# Supplementary material for: Machine Learning Reveals Microbial Taxa Associated with a Swim across the Pacific Ocean
Source: Biomedicines. 2024 Oct 11;12(10):2309. doi: 10.3390/biomedicines12102309 (PMC11504845; doi:10.3390/biomedicines12102309)
Supplement: Supplementary file 1 [file biomedicines-12-02309-s001.zip › biomedicines-3205895-supplementary.pdf]

Supplemental Figure Legend

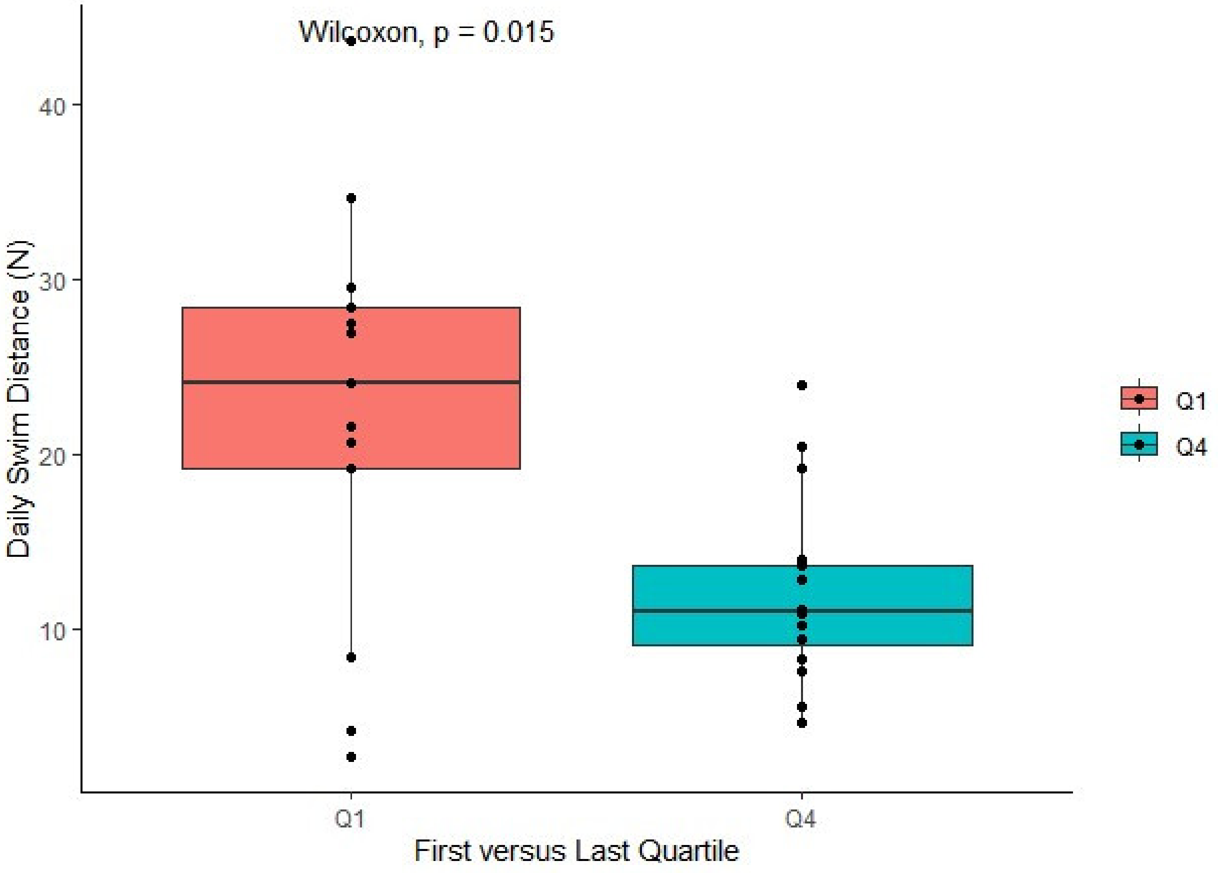

Scheme S1. First and Last Quartile of Daily Swim Distance. Box plots illustrating daily swim distance for the first (Q1) and last (Q4) quartiles.

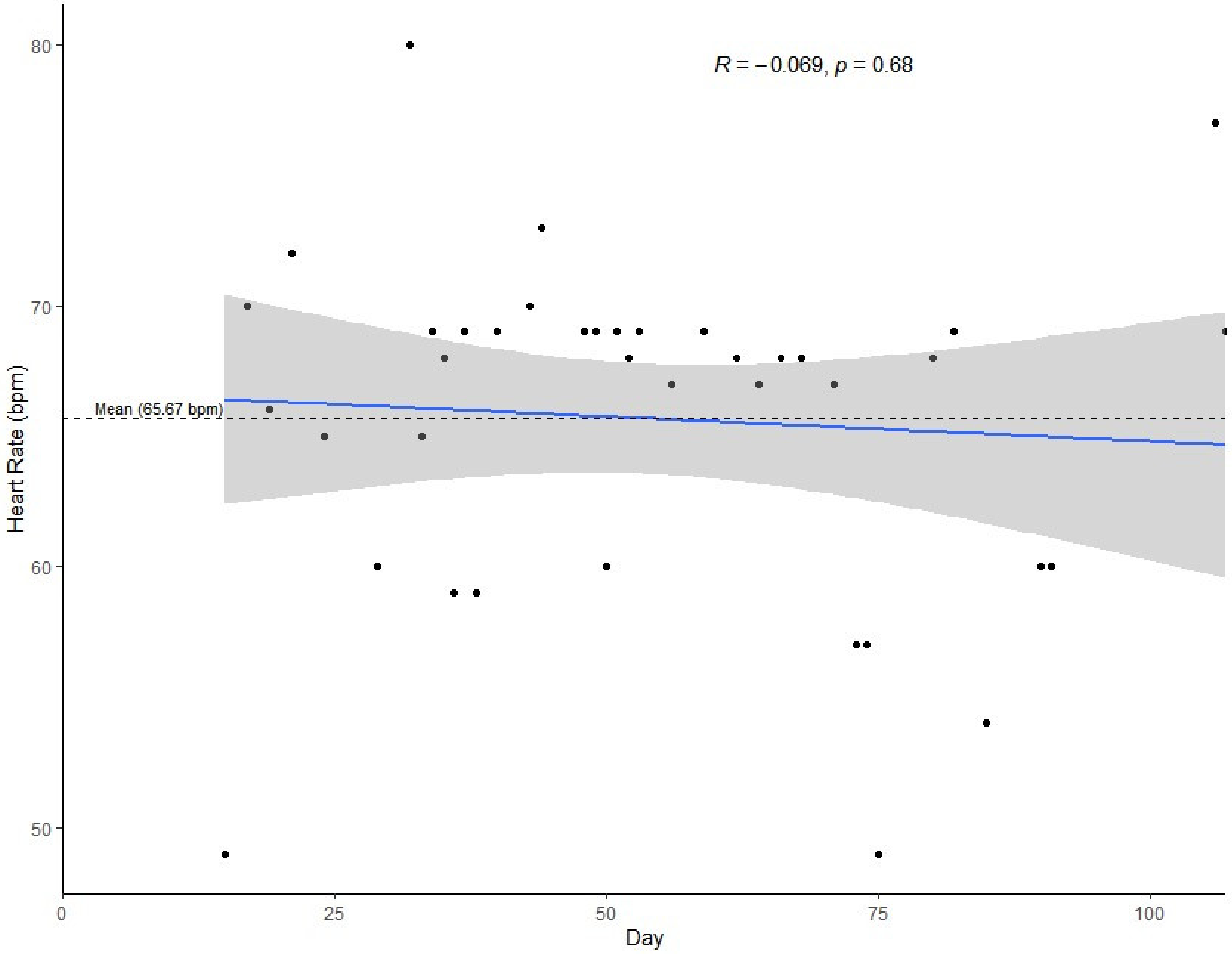

Scheme S2. Heart rate over time. A linear model shows the participant's resting heart rate over time.

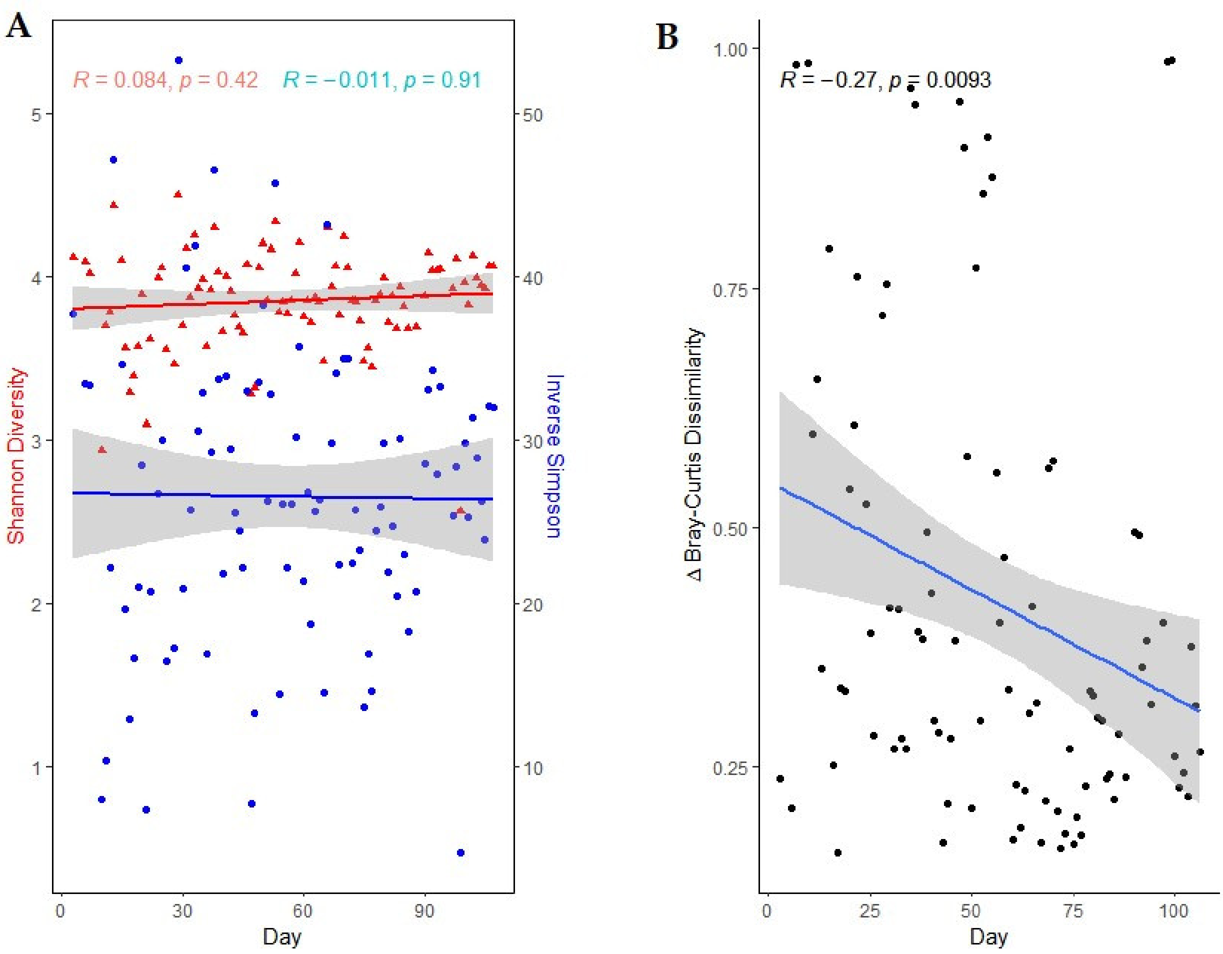

Scheme S3. Alpha and beta diversity over time. (A) Shannon diversity is represented in red triangles, while Inverse Simpson diversity is indicated in blue dots. The Shannon index mainly accounts for species richness and evenness, while the Simpson index emphasizes species dominance. (B) The Bray-Curtis dissimilarity quantifies the degree of dissimilarity between consecutive days by considering species abundance or presence/absence differences.

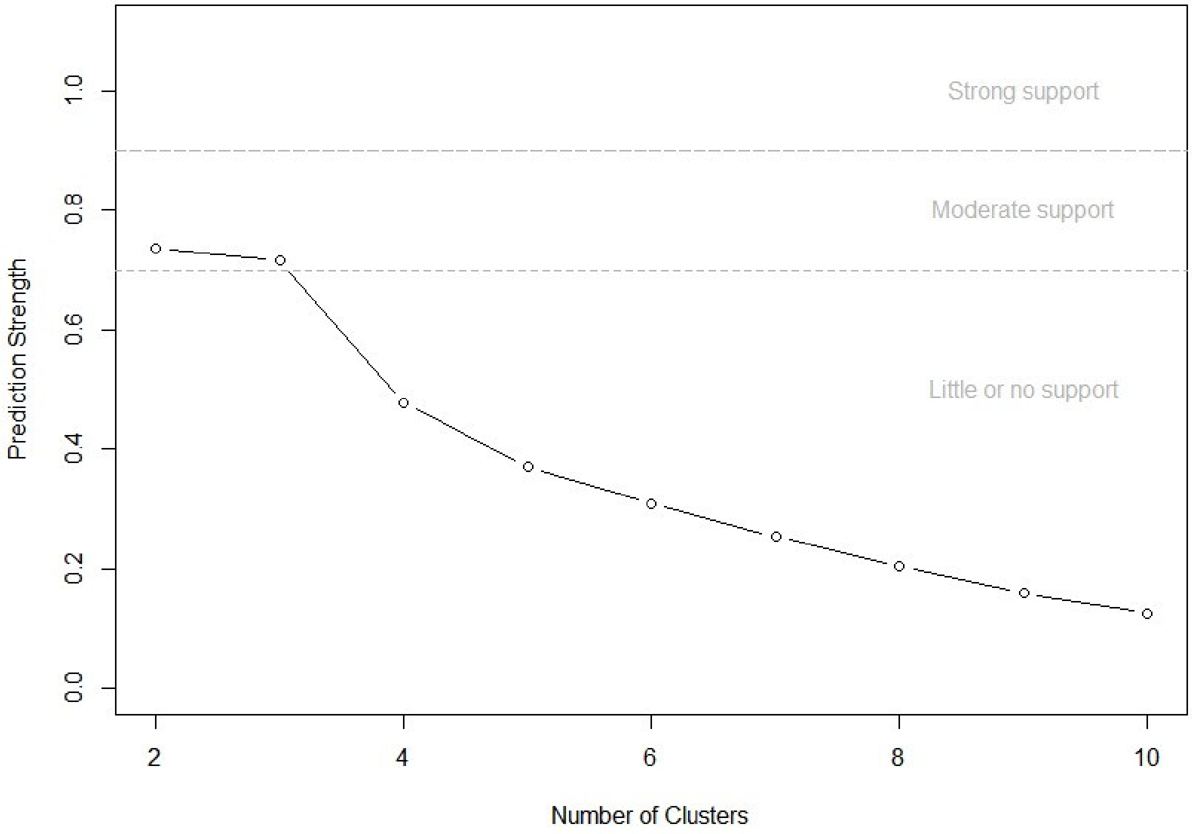

Scheme S4. Verification of K-Means Clusters. Using Bray-Curtis distances, over 70% of the variance in the data was explained by two clusters, exhibiting a moderate prediction strength.
